# Triangulating Qualitative Methods to Evaluate Survivorship Care

**DOI:** 10.1093/geroni/igaa057.2405

**Published:** 2020-12-16

**Authors:** Aaron Seaman, Seyedehtanaz Saeidzadeh, Emily Chasco, Sangil Lee, Nicholas Kendell, Heather Reisinger, Nitin Pagedar

**Affiliations:** 1 Carver College of Medicine, University of Iowa, Iowa City, Iowa, United States; 2 College of Nursing, University of Iowa, Iowa City, Iowa, United States; 3 College of Medicine, University of Iowa, Iowa City, Iowa, United States

## Abstract

With 16.9 million survivors in the US, survivorship is an increasingly important aspect of oncologic care. As the number increases, we need to provide evidence-based, standardized survivorship care, yet the evidence base is lacking and guidelines are variably implemented. This multi-sited study documented the survivorship care practices of five head and neck cancer (HNC) programs in order to identify survivorship care practices, provider preferences, practice variability, and the facilitators and barriers to effective survivorship care implementation. To ensure rich, contextual data, the study utilized multiple qualitative methods: 1) program characteristics questionnaire; 2) semi-structured interviews with providers involved in treatment and survivorship care, 3) on-site observation and clinic workflow mapping, and 4) collection of survivorship materials. Triangulating data collection provided evidence of potentially promising HNC survivorship care practices, aligned with the vision of comprehensive survivorship care, that can be used to evaluate practices and develop interventions on a larger scale.

